# Transcriptome Profiling of Pediatric Core Binding Factor AML

**DOI:** 10.1371/journal.pone.0138782

**Published:** 2015-09-23

**Authors:** Chih-Hao Hsu, Cu Nguyen, Chunhua Yan, Rhonda E. Ries, Qing-Rong Chen, Ying Hu, Fabiana Ostronoff, Derek L. Stirewalt, George Komatsoulis, Shawn Levy, Daoud Meerzaman, Soheil Meshinchi

**Affiliations:** 1 Center for Biomedical Informatics and Information Technology, National Cancer Institute, Rockville, MD, 20850, United States of America; 2 Fred Hutchinson Cancer Research Center, Seattle, WA, United States of America; 3 Hudson Alpha Institute for Biotechnology, Huntsville, AL, United States of America; Queen's University Belfast, UNITED KINGDOM

## Abstract

The t(8;21) and Inv(16) translocations disrupt the normal function of core binding factors alpha (CBFA) and beta (CBFB), respectively. These translocations represent two of the most common genomic abnormalities in acute myeloid leukemia (AML) patients, occurring in approximately 25% pediatric and 15% of adult with this malignancy. Both translocations are associated with favorable clinical outcomes after intensive chemotherapy, and given the perceived mechanistic similarities, patients with these translocations are frequently referred to as having CBF-AML. It remains uncertain as to whether, collectively, these translocations are mechanistically the same or impact different pathways in subtle ways that have both biological and clinical significance. Therefore, we used transcriptome sequencing (RNA-seq) to investigate the similarities and differences in genes and pathways between these subtypes of pediatric AMLs. Diagnostic RNA from patients with t(8;21) (N = 17), Inv(16) (N = 14), and normal karyotype (NK, N = 33) were subjected to RNA-seq. Analyses compared the transcriptomes across these three cytogenetic subtypes, using the NK cohort as the control. A total of 1291 genes in t(8;21) and 474 genes in Inv(16) were differentially expressed relative to the NK controls, with 198 genes differentially expressed in both subtypes. The majority of these genes (175/198; binomial test *p*-value < 10^−30^) are consistent in expression changes among the two subtypes suggesting the expression profiles are more similar between the CBF cohorts than in the NK cohort. Our analysis also revealed alternative splicing events (ASEs) differentially expressed across subtypes, with 337 t(8;21)-specific and 407 Inv(16)-specific ASEs detected, the majority of which were acetylated proteins (p = 1.5x10^-51^ and p = 1.8x10^-54^ for the two subsets). In addition to known fusions, we identified and verified 16 *de novo* fusions in 43 patients, including three fusions involving *NUP98* in six patients. Clustering of differentially expressed genes indicated that the homeobox (*HOX*) gene family, including two transcription factors (*MEIS1* and *NKX2-3*) were down-regulated in CBF compared to NK samples. This finding supports existing data that the dysregulation of *HOX* genes play a central role in biology CBF-AML hematopoiesis. These data provide comprehensive transcriptome profiling of CBF-AML and delineate genes and pathways that are differentially expressed, providing insights into the shared biology as well as differences in the two CBF subsets.

## Introduction

Acute myeloid leukemia (AML) is a hematopoietic malignancy defined by genetic (and epigenetic) alterations in hematopoietic stem or progenitor cells that lead to dysregulation of critical signal transduction pathways resulting in clonal expansion without complete differentiation. The genomic landscape of AML is under investigation. Distinct profiles have been discovered for different karyotypes and single-nucleotide polymorphisms (SNPs), revealing the heterogeneity and complexity of AML[1]. This genomic complexity leads to variability in responses to chemotherapy and disparate outcomes. Moreover, we and others have found age-dependent shifts in the genomic abnormalities of AML, some of which [2, 3] may contribute to differential outcomes observed in adult vs. pediatric AML[4]. Although these previous studies have helped us to better understand the correlation between genotypes and phenotypes in AML, a more detailed examination of defined molecular subgroups may yield another level of understanding, which is not readily attainable by examining more molecular diverse AML populations.

Cytogenetic alterations have been shown to play a critical role in the diagnosis of AML[1]. Fusions involving *RUNX1-RUNX1T1* and *CBFB-MYH11*, collectively referred to as core binding factor (CBF) AML, are one of the most frequent and most-studied genomic events in AML[5, 6]. Despite extensive studies into the biologic implications of these fusion transcripts and their use for risk stratification,[7, 8] knowledge of the presence of these fusions has not led to new targeted interventions. Further, despite the fact that t(8;21) and Inv(16) implicate CBFA and CBFB, respectively, and lead to similar clinical outcomes, potential mechanistic similarities and differences remain to be well defined.

RNA-seq for whole-transcriptome sequencing has become a powerful approach for studying mRNA transcripts[9, 10]. In contrast to traditional microarray methods, RNA-seq can identify *de novo* transcripts that are not represented in the reference genome (i.e., fusion genes)[11] while quantifying previously described reference transcripts[12] and identifying splicing alterations[13]. Recently, several adult AML studies using NGS technologies have been reported. The Cancer Genome Atlas (TCGA) Research Network[14] revealed the genomic and epigenetic landscapes of 200 adult *de novo* AML patients using whole-genome, whole-exome, RNA, and microRNA sequencing, along with DNA methylation studies. In addition, MacRae et al.[15] used RNA-seq to analyze 55 adult leukemia samples, identifying 119 genes whose expression is more consistent than the commonly used control genes across those leukemia samples. Lilljebjorn et al. [16] also used RNA-seq to identify fusion genes in adult leukemia patients. In contrast, the study of the pathogenesis of pediatric AML using NGS technologies is still in its earliest stages, and large studies have not extensively evaluated CBF-AML patients using this technology.

In this report, we use whole-transcriptome sequencing to interrogate the transcript profiles for pediatric CBF-AML, comparing these to transcripts from cases with normal karyotype. The results reveal that t(8;21) and Inv(16) translocations aberrantly impact a set of common genes and molecular pathways and there are unique gene-expression signatures, splicing differences, and fusions observed in the CBF subtype.

## Results

### Patient characteristics

This cohort includes specimens from 64 patients with *de novo* AML with either t(8;21), N = 17; Inv(16), N = 14; or normal karyotype (NK), N = 33 treated on Children’s Oncology Group (COG) pediatric AML clinical trials. Patients with NK were selected for those with and without FLT3/ITD Mutation (N = 14 and 19, respectively). Baseline characteristics of the patients are shown in S1 Table.

### RNA sequencing in pediatric AML samples

**Fig 1:**
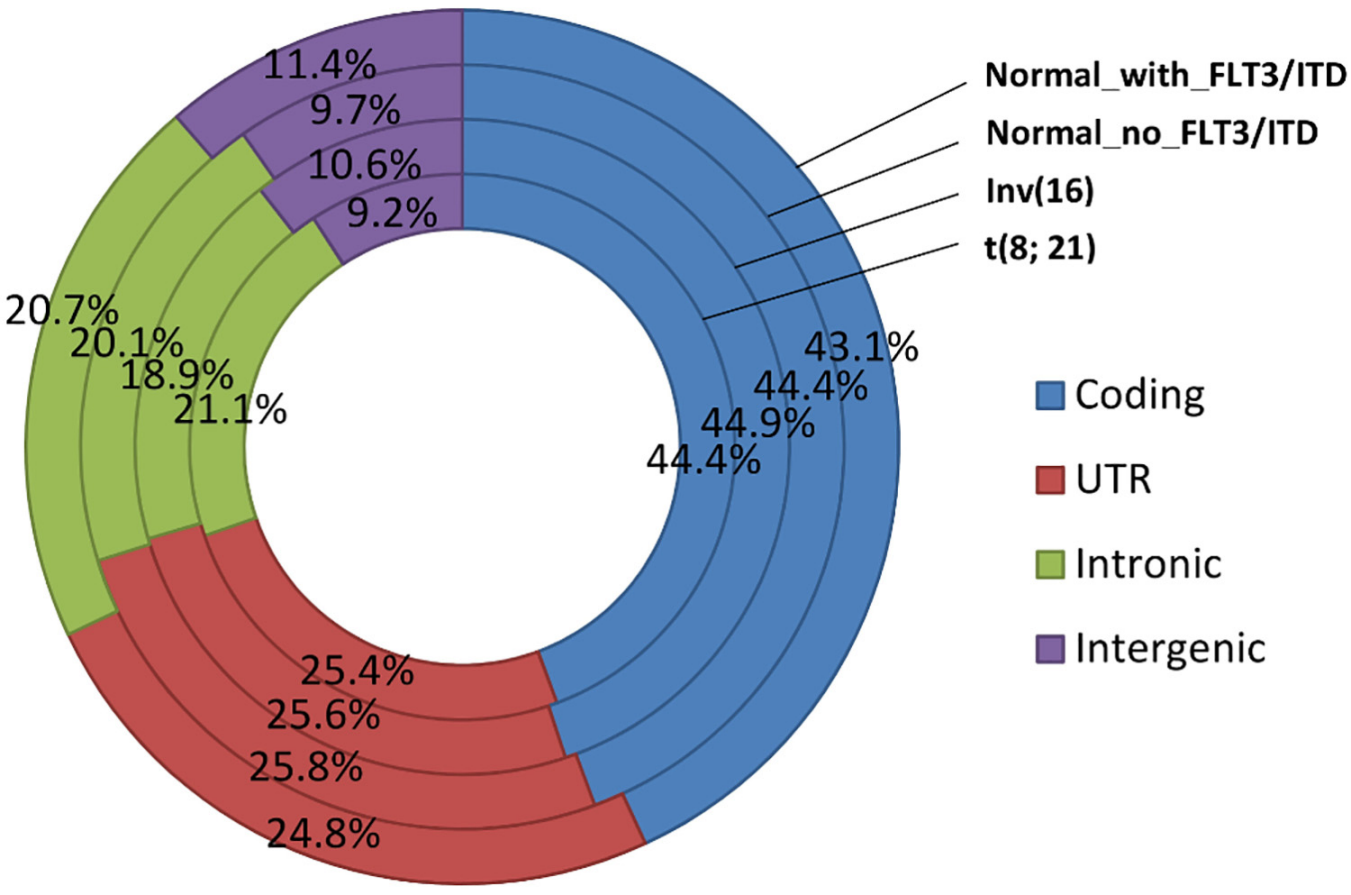
Distribution of aligned reads in the human genome (hg19).

RNA sequencing was performed using the Illumina platform for all 64 samples, with an average of 47 million (27,576,734–91,175,150) reads per sample. Ninety-six percent of these reads were mapped to the human reference sequence (hg19/NCBI Build 37) using the next-generation sequencing (NGS) aligner Novoalign (www.novocraft.com); ~26,000 RefSeq genes were covered by at least one read and ~16,500 RefSeq genes had RPKM (Reads Per Kilobase per Million mapped reads) ≥ 1 (S2 Table). Ninety percent of these mapped reads were located within gene regions, including coding, UTR, and intronic regions, and the distribution was very similar among different cytogenetic abnormalities (Fig 1).

### Identification of differentially expressed genes by RNA sequencing

**Fig 2:**
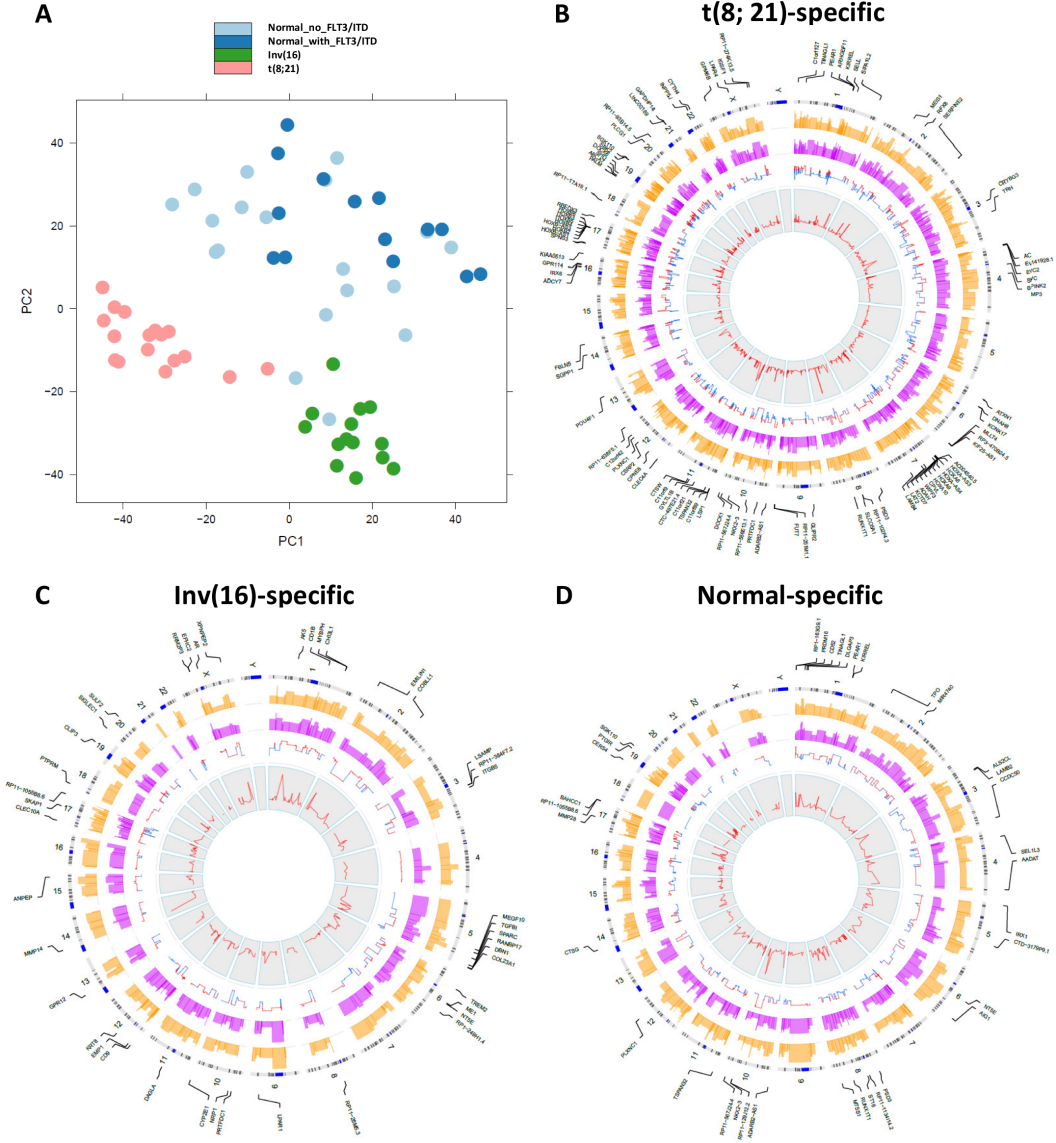
Differentially expressed genes characterize different cytogenetic abnormalities. (A) Principal component analysis for samples with different cytogenetic abnormalities. (B-D) Circular plots were drawn with the in-house software application OmicCircos[18] to represent the t(8;21)-specific, Inv(16)-specific, and normal-specific differentially expressed genes. The track from outside to inside are the symbols of differentially expressed genes with high significance (p-value < 1.0E-08); genome positions by chromosomes (black lines are cytobands); average expression level for the samples with specific cytogenetic abnormalities (yellow); average expression level for the remaining samples (pink); fold change (red: up-regulated; blue: down-regulated); and the p-values associated with the expression patterns between one subtype and the remaining samples.

In order to determine differential gene expression patterns specific to different cytogenetic categories, we performed principal component analysis (PCA) (Fig 2A). The PCA using all genes successfully separated out expression profiles for samples with Inv(16), t(8;21), or NK into three distinct clusters, suggesting that cytogenetic abnormalities profoundly affected gene-expression patterns. Two patients with NK had expression profiles that clustered with those with Inv(16). Closer examination of the two cases demonstrated the presence of *CBFB-MYH11* through fluorescence in situ hybridization (FISH) in 22% of the studied metaphases in one case. However, the second case did not show *CBFB-MYH11* fusions through FISH or real-time polymerase chain reaction (RT-PCR). The only fusion event shared by these two cases was the intergenic fusion *NDRG1-ST3GAL1*, which was also found in one t(8;21) sample and in one Inv(16) sample, but not in other NK samples.

To identify differentially expressed genes specific to each of the cytogenetic cohorts, we performed differential expression analysis using DESeq package[17], which uses a model based on the negative binomial distribution with variance and mean linked by local regression. Comparing t(8;21) samples with the remaining samples, a total of 827 t(8;21)-specific genes were found to be differentially expressed with an adjusted *p*-value (multiple testing using the Benjamini-Hochberg method) of less than 0.05 (Fig 2B). Among these, 365 genes were up-regulated, with the *RUNX1T1* gene most significantly up-regulated (p = 2.21x10^-31^; Table 1). RNA-seq reads were uniquely mapped into the entire coding regions of *RUNX1T1* for the 17 samples with t(8;21), with very few reads mapping to this gene in patients with Inv(16) or NK (S1 Fig). Additionally, 462 genes were down-regulated in samples with t(8;21), with the *RFX8* gene being the most under-expressed (p = 7.18 x 10^−20^). Eight of the top 20 under-expressed genes belonged to the *HOX* family (Table 1).

**Table 1:** Most significantly up- and down-regulated genes in the three cytogenetic categories.

t(8; 21)				Inv(16)				Normal			
Up-regulation		Down-regulation		Up-regulation		Down-regulation		Up-regulation		Down-regulation	
Gene	p-value	Gene	p-value	Gene	p-value	Gene	p-value	Gene	p-value	Gene	p-value
**RUNX1T1**	**2.21E-31**	**RFX8**	**7.18E-20**	**MMP14**	**7.06E-22**	**COL23A1**	**1.02E-09**	**NKX2-3**	**6.60E-17**	**ADARB2-AS1**	**7.85E-11**
**CTC-497E21.4**	**7.55E-22**	**TSPAN32**	**5.14E-19**	**AK5**	**5.32E-21**	**RP1-249H1.4**	**5.98E-09**	**RP11-129J12.2**	**1.73E-10**	**MMP28**	**5.70E-10**
**SIPA1L2**	**7.91E-19**	**MEIS1**	**7.02E-17**	**XPNPEP2**	**2.79E-16**	**RP11-1055B8.6**	**1.47E-08**	**RP11-1055B8.6**	**7.16E-10**	**RP11-1134I14.2**	**1.51E-09**
**TRH**	**3.55E-18**	**RP11-556E13.1**	**5.44E-16**	**EMILIN1**	**3.26E-16**	**RANBP17**	**3.05E-07**	**PRDM16**	**1.51E-09**	**KIRREL**	**2.42E-09**
**WIPF3**	**4.34E-14**	**HOXB2**	**8.00E-14**	**CHI3L1**	**9.33E-11**	**DBN1**	**8.50E-06**	**BAHCC1**	**2.42E-09**	**LAMB2**	**2.42E-09**
**POU4F1**	**9.74E-13**	**C11orf21**	**2.28E-13**	**CD9**	**2.44E-10**	**TANC2**	**2.08E-05**	**RP1-163G9.1**	**2.91E-09**	**DLGAP3**	**4.84E-09**
**PALM**	**1.06E-12**	**ARHGEF11**	**1.19E-11**	**SPARC**	**2.44E-10**	**DNAJC12**	**3.50E-05**	**TSPAN32**	**1.25E-07**	**CD52**	**6.48E-09**
**AC141928.1**	**2.37E-12**	**HOXB-AS1**	**2.77E-11**	**TGFBI**	**2.44E-10**	**CD59**	**5.15E-05**	**PLXNC1**	**2.86E-07**	**RUNX1T1**	**8.99E-09**
**PSD3**	**1.86E-11**	**AC004540.5**	**2.99E-11**	**LPAR1**	**4.57E-10**	**CYP7B1**	**5.39E-05**	**CTD-3179P9.1**	**5.31E-07**	**PEAR1**	**1.44E-08**
**IRX6**	**1.07E-10**	**LAT2**	**3.96E-11**	**ME1**	**1.18E-09**	**SPATA6**	**6.71E-05**	**SEL1L3**	**1.21E-06**	**AADAT**	**1.63E-08**
**GAPDHP14**	**1.24E-10**	**HOXB4**	**1.70E-10**	**GPR12**	**2.14E-09**	**NR6A1**	**7.13E-05**	**AIG1**	**2.17E-06**	**PSD3**	**2.58E-08**
**IGSF1**	**1.37E-10**	**CPVL**	**2.56E-10**	**COBLL1**	**2.67E-09**	**CTC-455F18.3**	**1.52E-04**	**CTSG**	**3.26E-06**	**CERS4**	**1.42E-07**
**SGPP1**	**1.55E-10**	**HOXA-AS4**	**3.69E-10**	**CLIP3**	**5.54E-09**	**RP11-480D4.3**	**1.72E-04**	**MIR4740**	**5.05E-06**	**RP11-567J24.4**	**1.11E-06**
**DOCK6**	**6.10E-10**	**HOXA10**	**5.79E-10**	**LSAMP**	**7.66E-09**	**PCNXL2**	**1.83E-04**	**RP11-1055B8.4**	**1.86E-05**	**ST18**	**1.62E-06**
**EVC2**	**1.24E-09**	**C1orf127**	**8.84E-10**	**CD1B**	**8.22E-09**	**LRRC37A16P**	**1.83E-04**	**TRGC1**	**2.27E-05**	**TPO**	**1.71E-06**
**SLCO5A1**	**1.32E-09**	**HOXA9**	**1.80E-09**	**EMP1**	**2.93E-08**	**BAHCC1**	**1.94E-04**	**OCLN**	**2.63E-05**	**SGK110**	**3.89E-06**
**PLCG1**	**1.67E-09**	**DOCK1**	**2.79E-09**	**CYP2E1**	**3.19E-08**	**NKX2-3**	**2.47E-04**	**BEND6**	**2.63E-05**	**ALS2CL**	**3.89E-06**
**GYLTL1B**	**2.35E-09**	**HOXB3**	**9.41E-09**	**NT5E**	**6.47E-08**	**RP5-862P8.2**	**2.47E-04**	**HOXA10**	**2.85E-05**	**CCDC50**	**5.05E-06**

Similarly, AML samples with Inv(16) displayed 279 genes that were differentially expressed as compared to the remaining samples, with 181 of these genes up-regulated and 98 down-regulated at the adjusted *p*-value of 0.05 (Fig 2C). Matrix metallopeptidase 14 (membrane-inserted) (*MMP14*) mRNA was most significantly up-regulated (p = 7x10^-22^). Conversely, collagen type XXIII, alpha 1 (*COL23A1*) mRNA was the most significantly down-regulated. Three hundred and eighty normal-specific genes were also found (Fig 2D), indicating a widespread presence of differentially expressed genes among different cytogenetic abnormalities.

### *RUNX1* binding sites were enriched in differentially expressed genes

*RUNX1* has shown to play a crucial role in haematopoiesis during embryonic development [18] and the two subunits of the core binding factors (CBFs), i.e., CBFA and CBFB, have been suggested to modify the transcriptional regulator functions of AML by either altering the normal *RUNX1* transcription program, interfering with the *RUNX1* assembly, or recruiting histone deacetylases and inhibiting the *RUNX1* activity [19–21]. To study the expression of the *RUNX1* targeted genes in the three cytogenetic categories, *RUNX1* ChIP-Seq data in the ME-1 cell line were analyzed [19](GEO accession number GSE46044). 34,654 peaks were identified using HOMER ChIP-Seq analysis package (http://homer.salk.edu/) and 11,844 out of 20,805 (56.9%) ensemble coding genes (GRCh37) were targeted by these ChIP-Seq peaks. Compared with the differentially expressed genes in the three cytogenetic categories, 72.8% of differentially expressed genes in the t(8;21) samples; 73.8% of differentially expressed genes in the Inv(16) samples and 69.0% of differentially expressed genes in the normal samples were targeted by these ChIP-Seq peaks. There is significant enrichment for the *RUNX1* binding sites in the differentially expressed genes in these three cytogenetic categories (Binomial test p-value is 2.1E-17, 7.1E-08 and 2.1E-05 respectively; S3 Table).

### Genes commonly expressed in CBF AML

**Fig 3:**
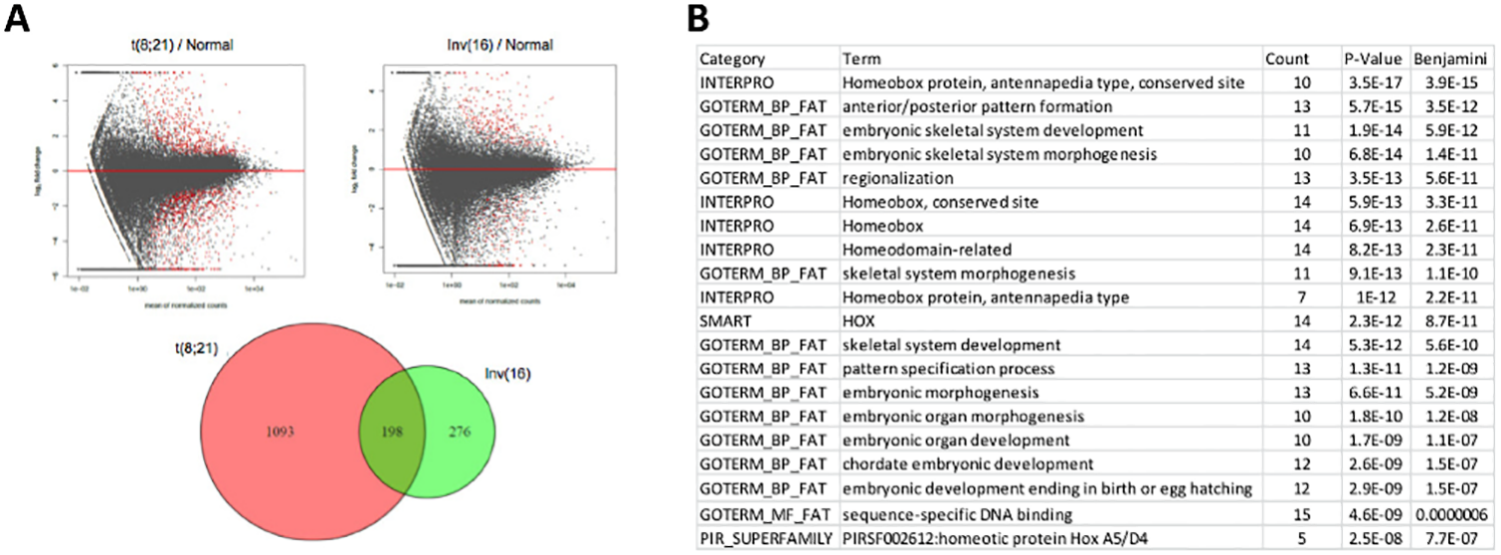
Commonly expressed genes in CBF AML. (A) Differentially expressed genes (red dots in the MA plot) in t(8;21) and Inv(16) vs. those with NK. 198 genes are shared in the two subtypes. (B) Gene Set Enrichment Analysis (GSEA) shows enriched functions for shared down-regulated genes between them.

Because those cases referred to collectively as CBF AML share a common biology, clinical presentation, and outcome, we inquired whether the two cohorts also shared an expression profile. To this purpose, we detected differentially expressed genes in t(8;21) and Inv(16) using NK cohort as the control. Of the total of 1567 genes that are differentially expressed in all CBF AML cases [1291 in t(8;21) and 474 in Inv(16)], compared to samples with normal karyotype (NK), 198 differentially expressed genes are shared by the two subtypes in CBF AML (Fig 3A): 87 of these genes are up-regulated, 88 are down-regulated in both subtypes (S4 Table), while another 23 genes have opposing expression profiles (down-regulated in one subtype but up-regulated in the other). More genes share expression profiles between these two subtypes (175 / 198; binomial test *p*-value < 10^−30^) than do not. Furthermore, the 88 shared down-regulated genes include many *HOX* genes and are enriched in genes involved in morphogenesis, specifically embryonic skeletal system development (Fig 3B). In contrast, no gene sets are significantly enriched for the 87 shared up-regulated genes.

### Expression profile in samples with normal karyotype (NK)

**Fig 4:**
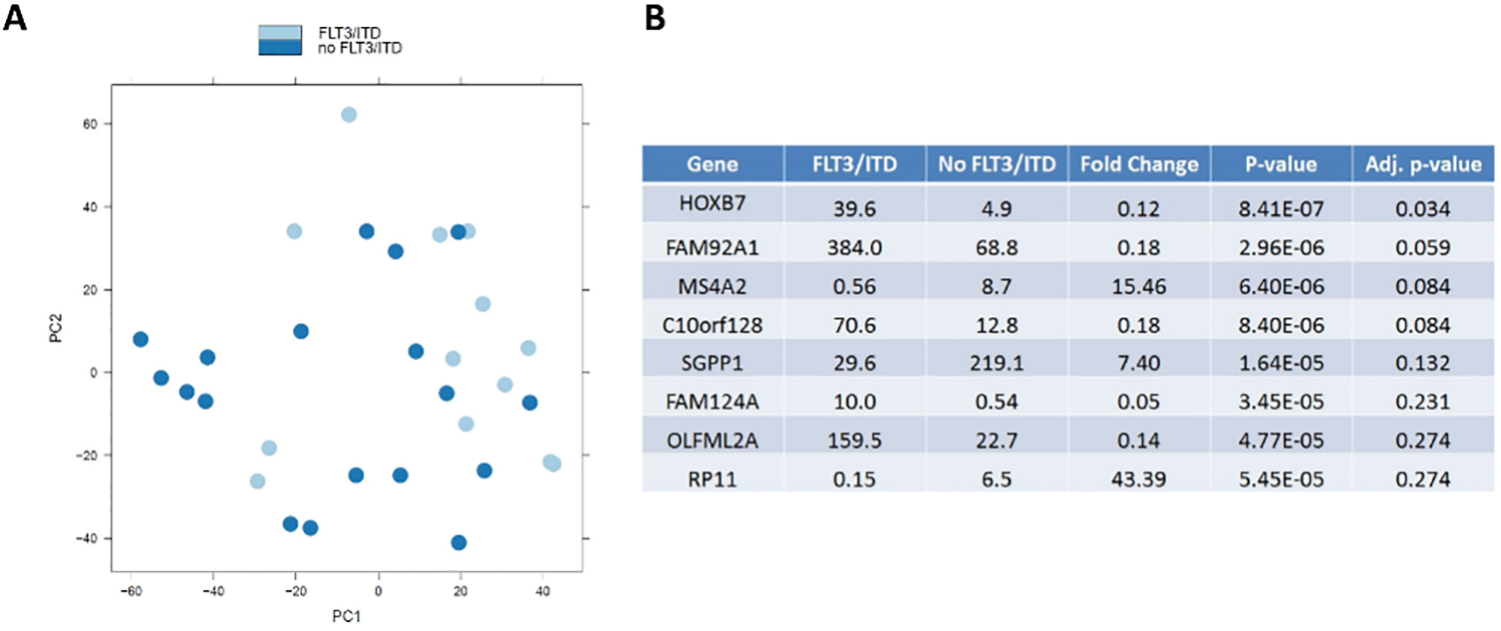
Comparison of expression profiles for those with and without *FLT3*/ITD mutation. (A) PCA for the two groups. (B) Genes with the most significant adjust *p*-value between the two groups.

Evaluation of distinct expression profiles of those with NK from those with t(8;21) or Inv(16) identified 175 significantly up-regulated and 205 down-regulated genes. We further studied the expression profiles for those with and without FLT3/ITD mutation in the NK cohort to determine whether FLT3/ITD mutation is associated with a specific expression pattern. Although the expression signature for those with NK was distinct from those with CBF AML, expression PCA failed to define a distinct expression profile for those with and without FLT3/ITD mutation (Fig 4A). *HOXB7* was the only gene whose expression was significantly associated with FLT3/ITD mutation (adjusted *p*-value 0.034) (Fig 4B) with an adjusted *p*-value < 0.05.

### Co-expressed genes define gene networks

**Fig 5:**
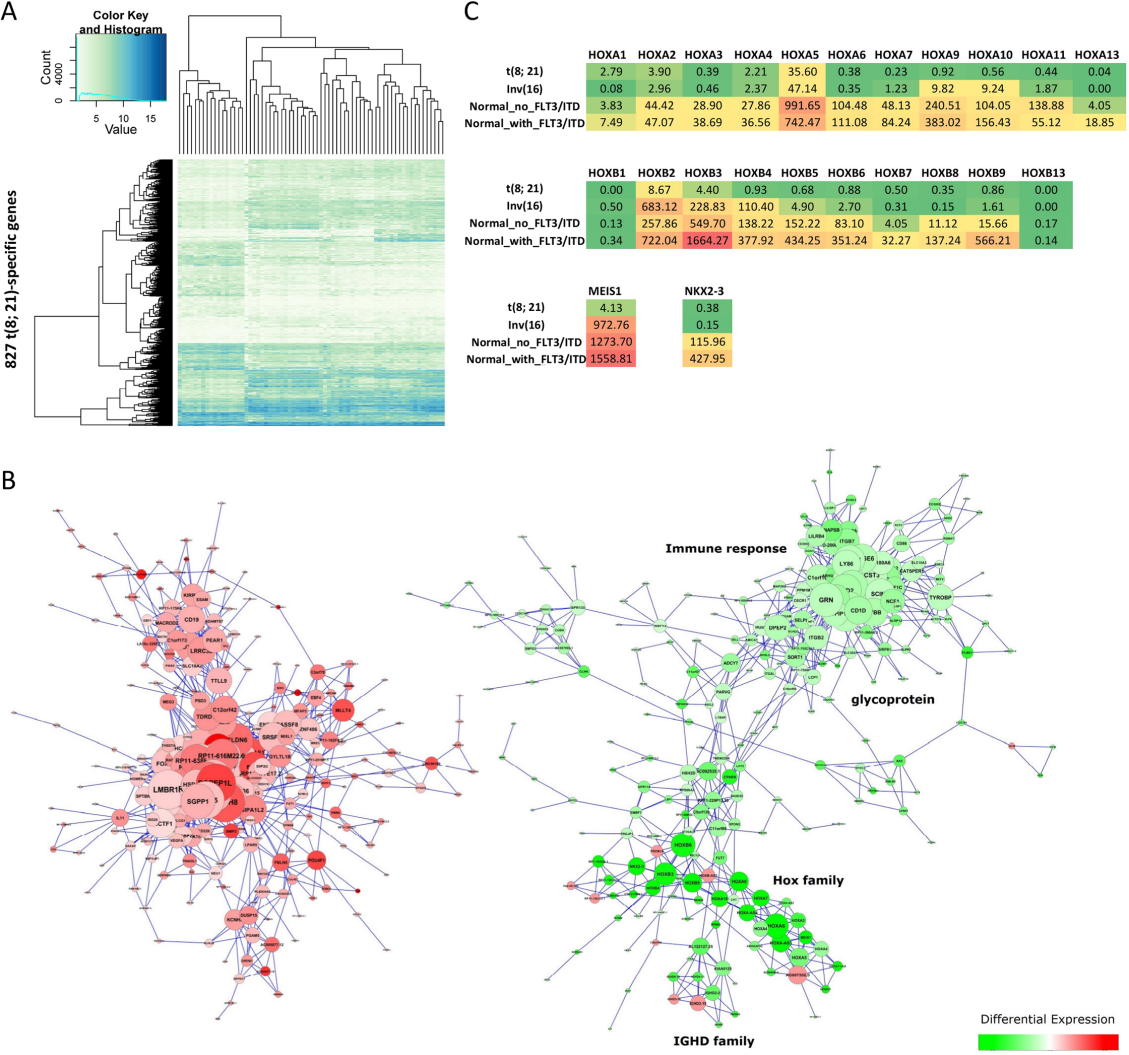
Co-expression of t(8;21)-specific genes. (A) Heatmaps showing the clustering of 827 t(8;21)-specific genes in 64 pediatric AML samples. (B) Co-expressed genes were determined based on the coefficient of determination (R2 > 0.6). The co-expression gene networks were generated using Cytoscape 2.8.3[19]. Node color is based on the fold change of the differentially expressed gene (red: up-regulated; green: down-regulated), and node size corresponds to the degree of the node (i.e., the number of edges incident to it). (C) Gene expression of the HOX gene family for three types of cytogenetic abnormalities, where NK is separated into two groups based on the mutation of FLT3/ITD.

Clustering of differentially expressed genes for each cytogenetic abnormality indicates the existence of co-expressed gene networks (Fig 5A, S2A and S2B Fig). To study the functionality of these networks, we calculated the correlation coefficient (R) for each pair of differentially expressed genes in each subtype and used Cytoscape[22] to identify co-expressed genes with R^2^ > 0.6 and to determine subsets of the co-expressed gene networks with specific molecular functions (Fig 5B, S2C and S2D Fig). Up-regulated genes and down-regulated genes were clustered into different groups for the t(8;21)-specific differentially expressed genes. A sub-group containing 39 genes, located in the group with down-regulated genes, is enriched in the homeobox (*HOX*) gene family (Fig 5B). The group includes two *HOX* gene clusters on human chromosomes 7p15 (*HOXA*) and 17q21 (*HOXB*), the *HOX* cofactor myeloid ecotropic viral integration site *1* (*MEIS1*) and the *NK2* homeobox 3 (*NKX2-3*). *MEIS1* is a common leukemic collaborator[23] and *NKX2-3* is a homeobox transcription factor[24]. All of these *HOX* genes were down-regulated in the samples with t(8;21) (Fig 5C). Most *HOX* genes were also down-regulated in the samples with Inv(16) except for *HOXB2*, *HOXB3*, *HOXB4* and *MEIS1*. Furthermore, although samples with NK had higher expression levels in the *HOX* gene family, most genes in the *HOXB* gene cluster and *NKX2-3* had even higher expression levels when they contained the FLT3-ITD mutation.

Several immunoglobulin-related gene families, including the Leukocyte Immunoglobulin-like Receptor (*LIR*) gene family and the Immunoglobulin Heavy (*IGH*) gene family, were also enriched in down-regulated genes for the t(8;21) AML samples, while no molecular processes or functions were enriched in up-regulated genes for the t(8;21) AML samples.

### Alternative splicing is common in AML and is affected by acetylation

In addition to evaluating differential expression patterns, we assessed alternative splicing among the three cytogenetic cohorts. We used the Multivariate Analysis of Transcript Splicing (MATS) application[25] to identify alternative splicing characteristic of samples from the cytogenetic subtypes. MATS is a computational tool that uses a statistical model with multivariate uniform prior to detecting differential alternative splicing events using RNA sequencing data. Five different alternative splicing events were detected by MATS, including skipped exon (SE), alternative 5’ splice site (A5SS), alternative 3’ splice site (A3SS), mutually exclusive exons (MXE), and retained intron (RI).

**Fig 6:**
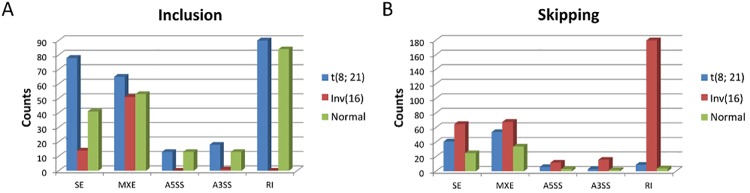
Alternative splicing for pediatric AML samples. Alternative splicing events detected by MATS for three cytogenetic abnormalities. Five different alternative splicing events were detected: skipped exon (SE), alternative 5’ splice site (A5SS), alternative 3’ splice site (A3SS), mutually exclusive exons (MXE) and retained intron (RI). All events were further separated into two groups based on whether the alternative exon was included (A) or skipped (B) in the samples.

In our study, 337 t(8;21)-specific, 407 Inv(16)-specific, and 272 NK-specific alternative splicing events were detected. Skipped exon (SE), mutually exclusive exons (MXE) and retained intron (RI) seemed to be the predominant alternative splicing events in pediatric AML samples (Fig 6A and 6B). Furthermore, MATS separated all alternative splicing events into inclusion or skipping groups based on whether the alternative exon was included or skipped in the samples. Samples with t(8;21) and NK tended to include the alternative exons (Fig 6A), while samples with Inv(16) were likely to skip them (Fig 6B). 216 genes were affected by t(8;21)-specific alternative splicing events (Most significant events involving genes including *RAB10*, *SERF2*, *HNRNPC*, *HNRNPD*, *HNRPDL*, *HINT1*, *NACA*, *PABPC1*, *RPL10*, *RPS12*, *RPS27*, *ARPC3*, *EIF1*, *STMN1*, and *ARPC4*). 233 and 158 genes were also affected by Inv(16)-specific and normal-specific alternative splicing events, respectively. Gene Set Enrichment Analysis (GSEA) indicated that the majority of the affected genes are acetylated proteins: 127 genes (59%; *p*-value = 1.5E-51) for t(8;21)-specific; 135 genes (58%; *p*-value = 1.8E-54) for Inv(16)-specific; and 98 genes (62%; *p*-value = 1.2E-42) for normal-specific, and are enriched in the KEGG pathway *ribosome*.

### Identification of fusion transcripts in pediatric AML samples

**Fig 7:**
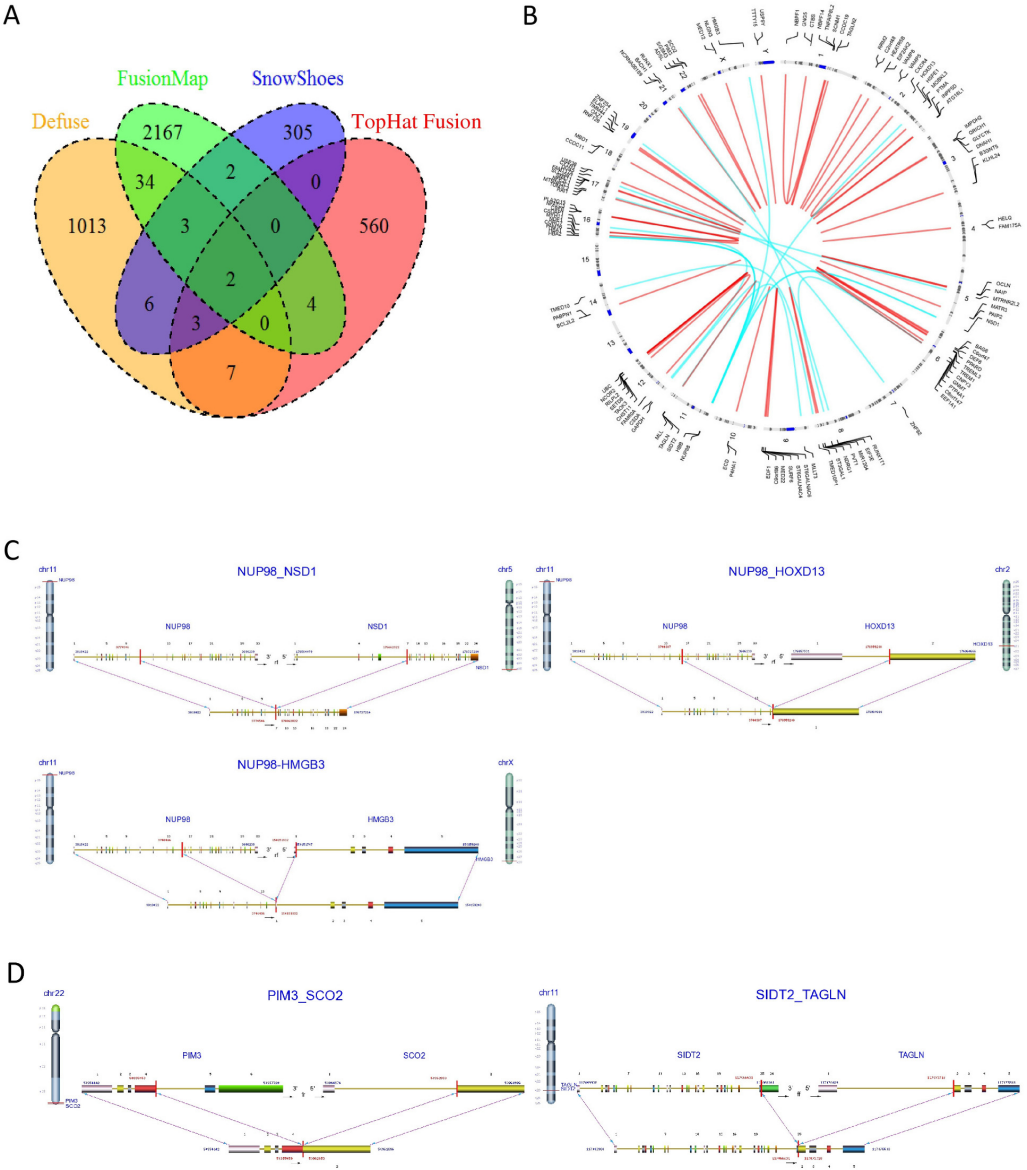
Identification of gene-fusion events in pediatric AML samples. (A) Gene-fusion events were detected using four gene fusion detection methods. (B) 69 putative fusion events shown in a circular plot. Red: intra-chromosomal fusion event; Blue: inter-chromosomal fusion event. (C) three fusion variants of NUP98. (D) Two in-frame fusions.

RNA sequencing has also been successfully used to identify gene-fusion events in cancers[26, 27], and many computational tools have been developed to detect these events[11, 28–30]. Most detection methods, however, have proved problematic because of high false-positive rates[31, 32]. We used four gene-fusion detection methods—Defuse[28], Tophat-Fusion[29], FusionMap[30] and Snowshoes-FTD[11] to identify gene-fusion events in our pediatric AML samples. The number of putative fusion events identified ranged from 300 to more than 2000 for each detection method, while only a few fusion events (2%–5%) overlapped between any two methods (Fig 7A). To reduce the high rate of false positives, only 69 putative fusion events (Fig 7B; S5 Table), identified by at least two detection methods or by one method with a ChimerDB hit[33], were used in our study. ChimerDB is a knowledgebase of fusion genes identified using bioinformatics analysis of transcript sequences based on various public resources, including GenBank, the Sanger Cancer Genome Project (CGP), OMIM, PubMed, and the Mitelman database. Fifty-one of the 69 putative fusion events (74%), were intra-chromosomal (Fig 7B), and the remaining 18 (26%) were found in inter-chromosomal junctions. Fifty-nine of the 69 identified fusions involved the coding regions of the affected gene (S4 Table). Eight putative fusion events were found in ChimerDB and six of them were previously reported in AML[34–37], suggesting that the combination of multiple gene-fusion detection methods and ChimerDB can accurately identify fusion events. The *CBFB-MYH11* fusion event was identified in 12 out of 14 samples with clinically annotated Inv(16) (Fisher's exact test; p-value = 1.25E-11). Closer interrogation of the two cases without the *CBFB-MYH11* fusion event demonstrated the presence of reads consistent with the fusion transcripts, but due to their low coverage, these cases did not meet the statistical threshold for identification. In addition, *RUNX1-RUNX1T1* transcript fusions were identified in all 17 samples with clinically annotated t(8;21) (Fisher's exact test; p-value = 2.23E-16), further suggesting that the identification of putative fusion events was accurate.

In addition to the known fusion transcript for the 31 patients with CBF AML (*CBFB-MYH11* and *RUNX1-RUNX1T1*), an additional 132 fusion transcripts (for 47 fusion events) were identified, including 21 inter- and 111 intra-chromosomal translocations. In 33 patients without known karyotypic alterations (NK), a total of 126 fusion transcripts (for 53 fusion events) was detected, including 30 inter- and 96 intra-chromosomal fusions. In total, 287 fusion transcripts were identified. These included intra-chromosomal junctions of three fusion variants of *NUP98* in 6 patients (*NUP98-NSD1*, N = 4; *NUP98-HOXD13*, N = 1; and *NUP98-HM*GB3, N = 1; Fig 7C). Frequent high-confidence in-frame fusions (i.e., *PIM3-SCO2*, *ADSL-SGSM3* and *SIDT2-TAGLN* (Fig 7D), as well as all *NUP98* variants) were confirmed by secondary methodology (PCR and Sanger sequencing).

There were 119 genes involved in the 69 fusion events and gene set enrichment analysis of these 119 genes using the Database for Annotation, Visualization, and Integrated Discovery (DAVID)[38] (Huang et al. 2009) indicated that these fusion genes are enriched in genes that code for proteins which are post-translationally modified by the attachment of at least one methyl, phosphate, or acetyl group.

## Discussion

Fusion transcripts resulting from genomic translocations between *RUNX1-RUNX1T1* in t(8;21) and *CBFB-MYH11* in Inv(16), collectively referred to as CBF AML, have similar clinical outcomes, but the similarities and differences between the two entities have not been studied in detail. We investigated the transcriptome profiles of specimens from children with AML characterized by t(8;21) or Inv(16), and also from a third subset of patients with normal karyotype (NK) in order to define transcript-expression patterns as well as isoforms and pathways that are differentially expressed in these genomic subsets and to delineate the similarities and differences in these groups of patients. Hundreds of differentially expressed genes were found in each cohort indicating a widespread presence of differential expression among different cytogenetic abnormalities. Using the NK cohort as our control, we established expression profiles for each of the two subtypes [i.e., t(8;21) and Inv(16)] in CBF AML to define genes whose expression patterns are shared or differ between the two subtypes. A large number of differentially expressed genes were identified for the subsets of t(8;21) and Inv(16) compared to the NK cohort. We then demonstrated that the majority of the shared differentially expressed genes (175 / 198; binomial test *p*-value < 10^−30^) between t(8;21) and Inv(16) are consistent in the two subtypes (genes are up- or down-regulated in both subtypes) and these genes are enriched for a number of *HOX* genes involved in morphogenesis, specifically the development of the embryonic skeletal system. Additionally, each of the subgroups were enriched for differentially expressed genes that contained *RUNX1* binding sites; 72.8% of genes in the t(8;21) samples; 73.8% of genes in the inv(16) samples and 69.0% of genes in the normal samples.

Besides karyotype-specific expression patterns, alternative splicing patterns among the cytogenetic cohorts were assessed. We identified a large number of specific alternative splicing events associated with each cytogenetic subset. Our data demonstrated that several types of splicing exist in our cohorts, with skipped exon (SE), mutually exclusive exons (MXE), and retained intron (RI) predominating. This suggests that splicing patterns in AML could be karyotype-specific. Different splicing events can modulate gene function by introducing or deleting functional gene domains or silencing genes by causing frame-shifts or introducing early-termination codon. Defining the functional isoforms that would be translated from those destined for nonsense-mediated decay is likely to prove critical in distinguishing biologically significant spliced-gene products from non-functional ones. Additionally, we demonstrated that the protein products of a significant majority of alternatively spliced genes are determined to be substrates of post-translational acetylation. Previous studies have demonstrated that acetylation strongly influences alternative splicing[39, 40], implicating acetylation in the karyotype-specific differential expression of alternative splicing. Gene set enrichment analysis of the genes altered by splicing indicated varied members of the KEGG pathway *ribosome* are involved. This is consistent with previous studies that show the fusion proteins, *RUNX1-RUNX1T1* and *CBFB-MYH11*, to regulate ribosomal RNA transcription [41, 42]. The dysregulation of these processes is thought to aid in the fusion proteins’ role in altering differentiation, proliferation and disrupting normal hematopoiesis.

We further assessed the presence of fusion transcripts found in our study population. Given the high false-positive rate for most current gene-fusion detection methods[10, 31], our integration of four distinct gene-fusion detection methods with ChimerDB, a knowledgebase of fusion genes, allowed us to identify fusion transcripts more accurately than is possible with approaches that use a single method. (We accurately identified 29 out of 31 cases for those known fusion events (*CBFB-MYH11* and *RUNX1-RUNX1T1*) in CBF AML.) In addition to identifying the 29 known fusion transcripts, we detected an additional 258 fusion transcripts (67 fusion events in 115 genes). Fusions of high interest include those involving *NUP98*, a nucleoporin gene that encodes a building block of the nuclear pore complex which mediates the transport of mediators of cellular function across the nuclear membrane. Three fusion variants of *NUP98* were identified (*NUP98-NSD1*, *NUP98-HOXD13*, and *NUP98-HMGB3*). *NUP98-NSD1* has been shown to co-occur with the mutation of FLT3/ITD and to be strongly associated with adverse outcomes[43], though *NUP98-HOXD13* and *NUP98-HMGB3* are less-known variants whose prevalence and clinical implications are yet to be determined. Further, we identified a large number of intra-chromosomal fusions whose true functionality needs be evaluated. Some of these fusions may result from transcriptional read-throughs that may or may not be functionally significant. Studies of the protein products and functional significance of these lesions is ongoing. Our study also indicates that the identified fusion transcripts are enriched in genes that encode proteins undergoing post-translational modification by acetylation, methylation, or phosphorylation, suggesting potential functional implications. A previous study has shown that chromatin proteins and metabolic enzymes are highly represented in acetylated, methylated, or phosphorylated proteins[44], suggesting that gene fusions may profoundly affect gene expression and metabolism in pediatric AML subtypes.

This study also highlights the significance of homeobox (*HOX*) genes in CBF AML. Dysregulation of *HOX* genes contributes to the perturbation of normal hematopoiesis[45, 46], and the overexpression of *HOX* genes in hematopoietic cells can contribute to leukemogenesis[47, 48]. Our results demonstrate that the expression levels of all *HOX* genes were down-regulated in the t(8;21) subtype, whereas in contrast, four *HOX* genes (*HOXB2*, *HOXB3*, *HOXB4 and MEIS1*) had much higher expression levels in the Inv(16) subtype than that in the t(8;21) subtype. The potential implication of differential *HOX* expression in CBF AML subtypes may cooperate with the leukemogenic potential of the two fusion events (*CBFB-MYH11* and *RUNX1-RUNX1T1*)[49]. Furthermore, given our observations of high *HOX* expression in patients with FLT3/ITD mutation and previous reports of association of elevated *HOX* expression with adverse outcomes[8, 50], the hypothesis that *HOX* expression may mediate the evolution of resistance should be considered.

This study provides comprehensive transcriptome profiles for CBF AML subtypes alpha [t(8;21)] and beta [Inv(16)]. It delineates differential gene-expression profiles, transcript splice isoforms, and fusion transcript profiles for CBF AML subtypes, and it also identifies specific genes and pathways that may provide targets for therapeutic intervention.

## Materials and Methods

### Pediatric AML samples

64 diagnostic samples derived from either bone marrow (n = 59) or peripheral blood (n = 5) were used in this study. All samples were obtained by written consent from the parents/guardians of minors from three consecutive Children’s Oncology Group clinical trials (CCG-2961, AAML-03P1, and AAML-0531). The Institutional Review Board at Fred Hutchinson Cancer Research Center has reviewed and approved this study. It is filed under protocol 1642 (Biology of the Alterations of the Signal Transduction Pathway in Pediatric Cancer), IR File #5236. Collectively, the percentage of leukemic blasts in the samples is very high with a median of 77.5% (range 40–100%). Age range is 0.83–20.82 years with a median of 12.29 years. Males represent 42 out of 64 (66%) patients.

### RNA preparation and sequencing

Genetic material from AML specimens was extracted using AllPrep DNA/RNA Mini Kits (Qiagen, Valencia, CA). At Hudson Alpha Institute (Huntsville, AL), 1 μg of high-quality total RNA was used for the conversion of mRNA into a cDNA library of template molecules based on mRNA capture with poly(T) magnetic beads, fragmentation, and reverse transcription to first-strand cDNA with reverse transcriptase and random primers using Illumina's TruSeq RNA Sample Prep kit (Illumina, San Diego, CA) according to the manufacturer's instructions. After adaptor ligation, each cDNA library was purified and enriched by PCR amplification; the final average fragment size, including adaptors, was 280 bases. Each library was then subjected to 50-cycle paired-end sequencing on the Illumina HiSeq, with four samples multiplexed into each flow cell lane.

### Alignment of RNA-sequencing reads to the human genome

Paired-end RNA-sequencing reads were aligned to the human reference genome (hg19/NCBI Build 37). Both the human reference genome and the splicing junction sequences were combined to form the reference sequences using the USeq MakeTranscriptome program[51], and RNA-sequencing reads were aligned to the whole genome and splice junctions using Novoalign (Novocraft 2010). Novoalign used a structural-variation penalty to determine whether paired-end reads should be reported when they did not form proper fragments. Finally, the aligned reads were sorted and indexed using SAMTools[52] and were stored in the SAM/BAM format. All BAM files have been deposited at The database of Genotypes and Phenotypes (dbGaP, http://www.ncbi.nlm.nih.gov/gap) under substudy, phs000465.v10.p3, TARGET: Acute Myeloid Leukemia (AML).

### Identification of differentially expressed genes

Aligned reads were annotated using the HTSeq package (http://www-huber.embl.de/users/anders/HTSeq/). We used the HTSeq-count program to calculate the number of reads mapped in each gene based on the Homo_sapiens.GRCh37.69.gtf annotation file downloaded from ensembl.org. A union overlap resolution mode was used to remove ambiguous reads. DESeq[17] was used to calculate the *p*-value among samples with different cytogenetic abnormalities for each gene. We applied the Benjamini-Hochberg procedure [53] to correct multiple testing and reported genes with an adjusted *p* value < 0.05 as differentially expressed genes (S6 Table).

### Gene set enrichment analysis and visualization

We used DAVID[38] to perform gene set enrichment analysis (GSEA) in order to associate molecular functions with the set of differentially expressed genes as well as with sets of alternative splicing and fusion genes. Furthermore, we used OmicCircos[54] and Cytoscape[22] to visualize the results of the analysis.

### Detection of alternative splicing among different cytogenetic abnormalities

We used the MATS program to detect five distinct alternative splicing events. Putative alternative splicing events were identified from the RNA-sequencing data using the annotation file that HTSeq had downloaded from ensembl.org. TopHat[55] was used to identify alternative splicing events, MATS was used to calculate the *p*-value for each alternative splicing event, and the false-discovery rate (FDR) control was applied to find differential alternative splicing events among samples with distinct cytogenetic abnormalities (S7 Table).

### Identification of fusion events

Four gene-fusion detection methods—Defuse[28], Tophat-Fusion[29], FusionMap[30] and Snowshoes-FTD[11] were used to identify gene-fusion events in the pediatric AML samples. Fusion events identified by more than one methods were chosen as putative fusion events (S8 Table). Moreover, a knowledgebase of fusion genes, ChimerDB[33], was used to include those fusion events, which were only detected by a gene-fusion detection method. Visualization of fusion events was created using an in-house Perl program, which is based on the GD graphics library and uses UCSC hg19 known gene as gene and exon reference.

### RT-PCR validation for putative fusion events

RNA was reverse-transcribed using Thermo Scientific’s Maxima H Minus First Strand cDNA Synthesis Kit (Thermo Fisher Scientific, Pittsburgh, PA). The resulting cDNA was used in PCR amplification of fusion junctions with primers listed in S9 Table. Fusion transcripts were verified by Sanger sequencing.

## Supporting Information

S1 Fig An example showing the differential expression of genes between samples with different cytogenetic abnormalities. RNA-seq reads were mapped to the region of *RUNX1T1* for 64 pediatric AML samples (red: high read density; green: low read density).
(TIF)

S2 Fig Co-expression of Inv(16)-specific and normal-specific genes. (A-B) Heatmaps showing the clustering of differentially expressed genes among 64 pediatric AML samples for Inv(16)-specific and normal-specific differentially expressed genes. (C-D) Co-expression gene networks for Inv(16)-specific and normal-specific differentially expressed genes. Co-expressed genes were determined based on the coefficient of determination (R^2^ > 0.6). The co-expression gene network was generated using Cytoscape 2.8.3 (Smoot et al. 2011). Node color is based on the fold change of the differentially expressed gene (red: up-regulated; green: down-regulated) and node size corresponds to the degree of the node (the number of edges incident to the node).
(TIF)

S1 Table Baseline characteristics of 64 pediatric AML patients. (XLSX)

S2 Table Summary of mapping data for 64 pediatric AML samples generated by RNA sequencing. (XLSX)

S3 Table List of Genes with RUNX1 binding sites in each Cytogenetic Group. (XLS)

S4 Table Shared down-regulated and up-regulated genes in CBF AML vs. those with normal karyotype (NK). (XLSX)

S5 Table List of 69 putative fusion events. (XLSX)

S6 Table Differential expression analysis using DESeq. (XLSX)

S7 Table Multivariate Analysis of Transcript Splicing (MATS). (XLS)

S8 Table Gene Fusion events Identified by 2 or more detection methods, or 1 detection method + ChimerDB. (XLSX)

S9 Table List of fusion primers. (XLSX)
