# Supplementary figures and images for: Transcriptome Profiling of Pediatric Core Binding Factor AML
Source: PLoS One. 2015 Sep 23;10(9):e0138782. doi: 10.1371/journal.pone.0138782 (PMC4580636; doi:10.1371/journal.pone.0138782)

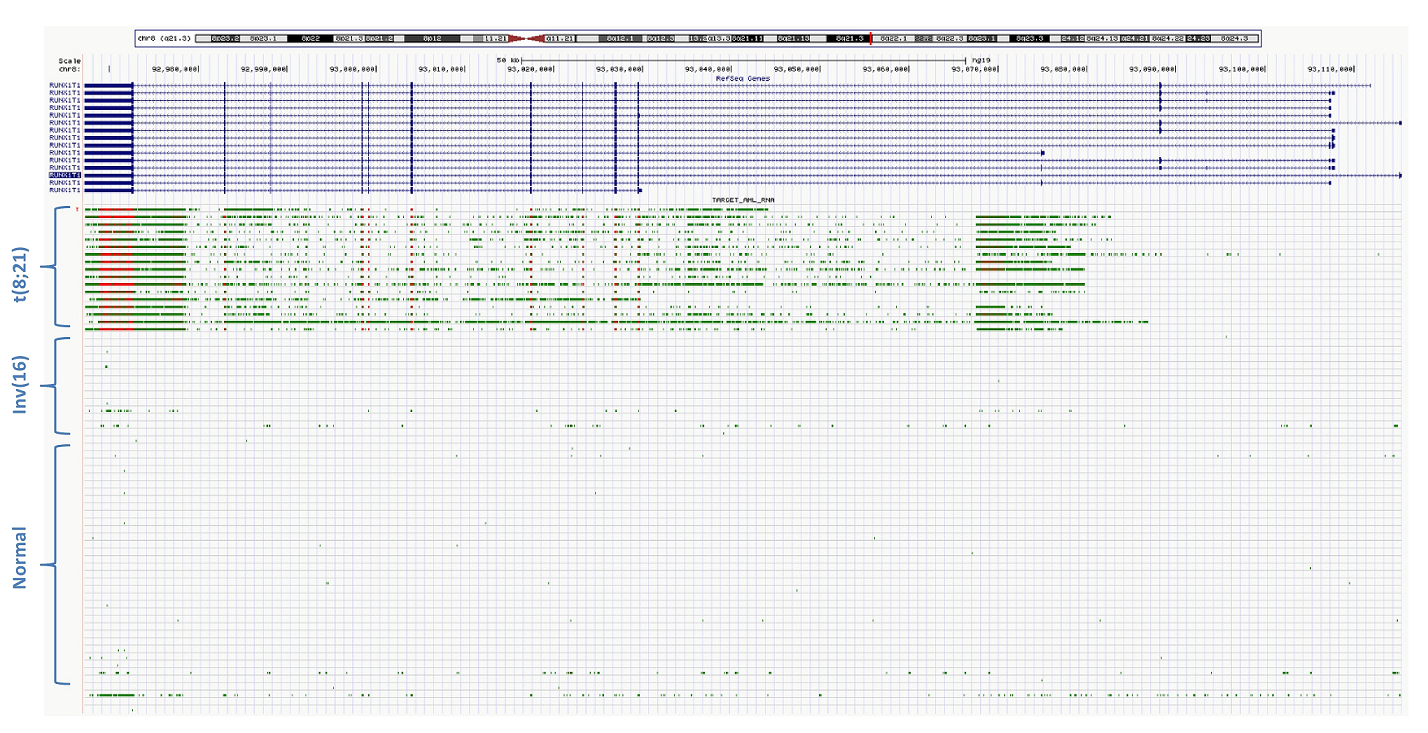

Supplement: S1 Fig — RNA-seq reads were mapped to the region of RUNX1T1 for 64 pediatric AML samples (red: high read density; green: low read density). (TIF) [file pone.0138782.s001.tif]

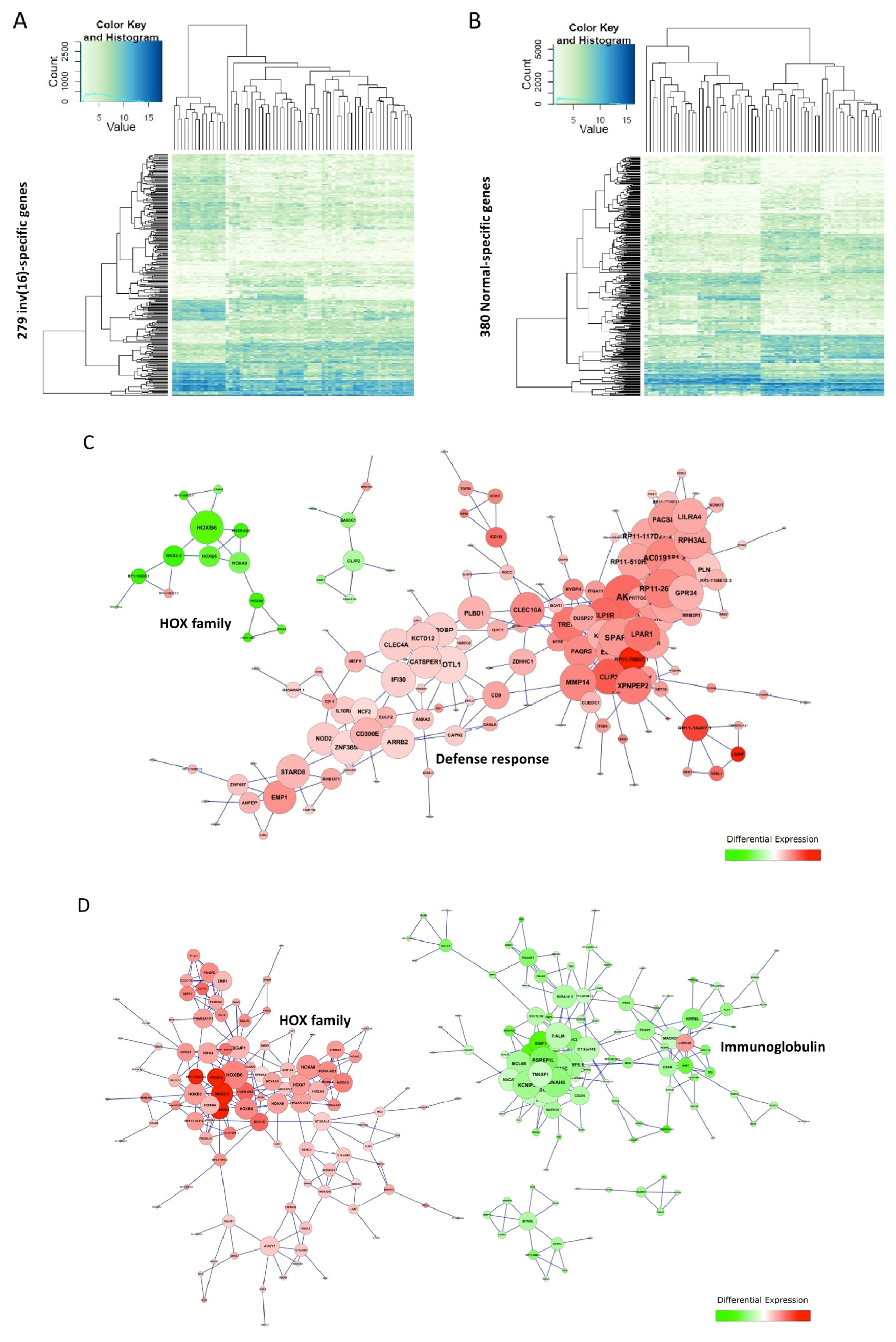

Supplement: S2 Fig — (A-B) Heatmaps showing the clustering of differentially expressed genes among 64 pediatric AML samples for Inv(16)-specific and normal-specific differentially expressed genes. (C-D) Co-expression gene networks for Inv(16)-specific and normal-specific differentially expressed genes. Co-expressed genes were determined based on the coefficient of determination (R2 > 0.6). The co-expression gene network was generated using Cytoscape 2.8.3 (Smoot et al. 2011). Node color is based on the fold change of the differentially expressed gene (red: up-regulated; green: down-regulated) and node size corresponds to the degree of the node (the number of edges incident to the node). (TIF) [file pone.0138782.s002.tif]
